# Clinical report of a neonate carrying a large deletion in the 10p15.3p13 region and review of the literature

**DOI:** 10.1186/s13039-021-00546-1

**Published:** 2021-05-28

**Authors:** Qiao-Yan Shao, Pei-Lin Wu, Bi-Yun Lin, Sen-Jing Chen, Jian Liu, Su-Qing Chen

**Affiliations:** Department of Pediatrics, The First Affiliated Hospital of Fujian Medical University, Chazhong Road 20, Taijiang District, Fuzhou, 350004 Fujian China

**Keywords:** 10p15.3 microdeletion syndrome, HDR syndrome, DiGeorge critical region 2, *ZMYND11*, *GATA3*

## Abstract

**Background:**

Terminal deletion of chromosome 10p is a rare chromosomal abnormality. We report a neonatal case with a large deletion of 10p15.3p13 diagnosed early because of severe clinical manifestations.

**Case presentation:**

Our patient presented with specific facial features, hypoparathyroidism, sen sorineural deafness, renal abnormalities, and developmental retardation, and carried a 12.6 Mb deletion in the 10p15.3 p13 region. The terminal 10p deletion involved in our patient is the second largest reported terminal deletion reported to date, and includes the *ZMYND11* and *GATA3* genes and a partial critical region of the DiGeorge syndrome 2 gene (*DGS2*).

**Conclusion:**

On the basis of a literature review, this terminal 10p deletion in the present case is responsible for a specific contiguous gene syndrome. This rare case may help the understanding of the genotype–phenotype spectrum of terminal deletion of chromosome 10p.

## Background

Terminal deletion of chromosome 10p is a rare chromosomal disorder. The haploinsufficiency in the distal region of 10p15.3 is responsible for 10p15.3 microdeletion syndrome (OMIM 608,668), characterized by specific facial features, neuropsychiatric retardation, and autism [1]. Additionally, hypoparathyroidism, deafness, and renal abnormalities (HDR syndrome; OMIM 146,255) occurs in patients with a haploinsufficiency of 10p14 in the distal region [2, 3], while deletions in a critical region of the DiGeorge syndrome 2 (DGS2; OMIM 601,362), located at the proximal region of 10p14-p13, are associated with congenital heart defects, thymus hypoplasia, and T cell defects [1].

Here, we report a Chinese infant showing specific facial features, congenital hypoparathyroidism, sensorineural hearing loss, absence of the right kidney, a sacrococcygeal mass, a right frontal cyst, and psychomotor retardation. The patient has a 12.6 Mb deletion in chromosome10p15.3p13. We summarized the clinical characteristics and laboratory data, in addition, we compared present case with previously published cases with terminal 10p deletions. Our data indicate that 10p15.3 microdeletion syndrome and HDR syndrome are associated with the partial monosomy 10p.

## Case presentation

A male infant was born by cesarean delivery at 36 weeks secondary to fetal heart deceleration. His birth weight was 2300 g (10th–50th centile), and his length and head circumference were within the normal ranges. He was pale, weak, and apneic at birth, and required positive pressure ventilation in the delivery room. His Apgar scores were 7, 8, and 8 at 1, 5, and 10 min, respectively. He was transferred to the neonatal department for respiratory support shortly after birth. There was no family history of developmental delay or inherited disorders, and his parents were non-consanguineous.

**Fig. 1:**
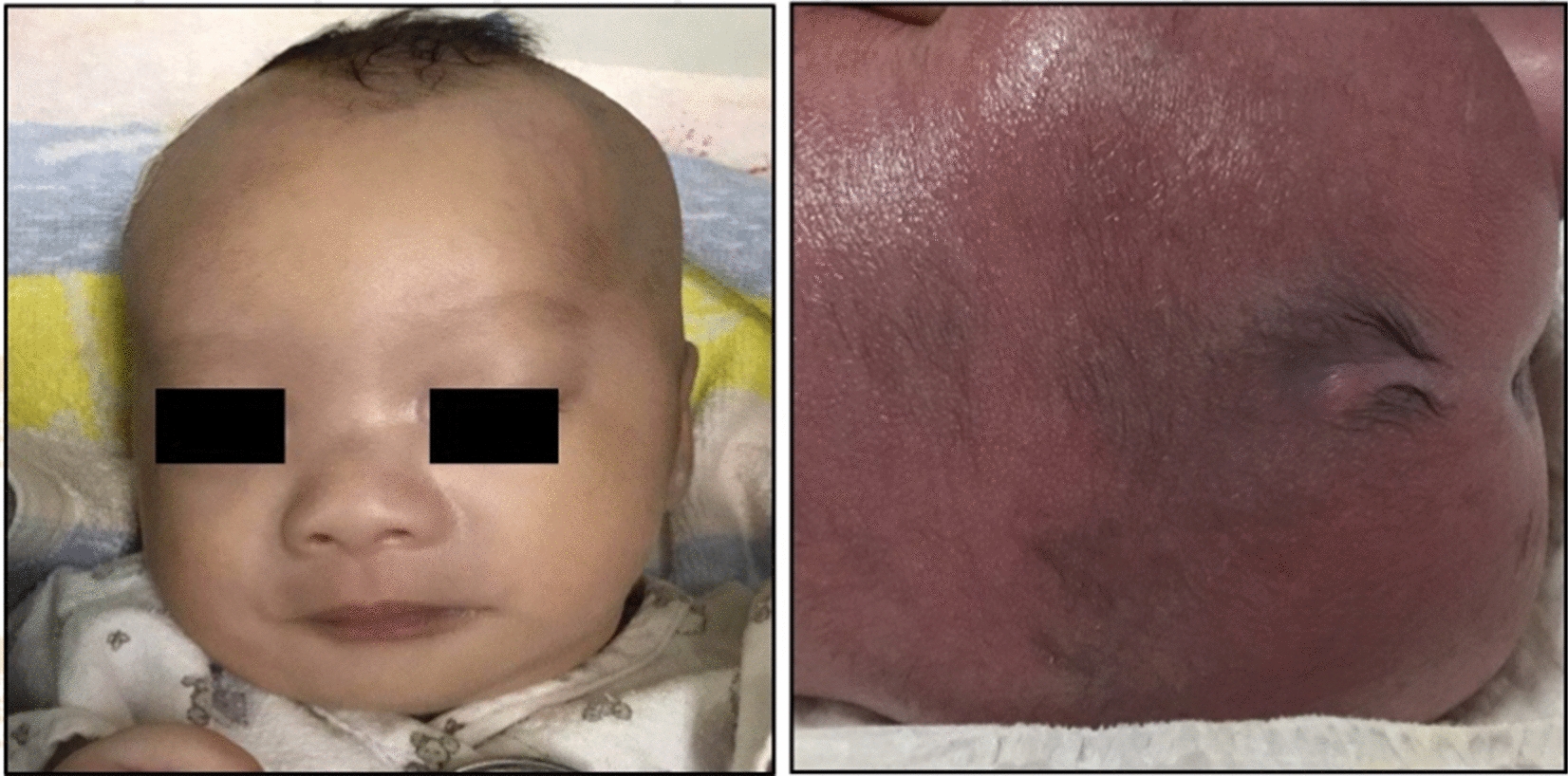
The patient has a wide ocular distance, low-set ears, and an abnormal sacrococcygeal mass

Clinical examination revealed an abnormal appearance (Fig. 1) including a prominent forehead, broad nasal bridge, small, low-set ears, micrognathia, and a sacrococcygeal mass. His anterior fontanelle was 3.5 × 3.0 cm, and cranial and sagittal sutures were approximately 1.0 cm. He also showed bilateral cryptorchidism. No deformity was found in the bones of his limbs, neck, chest, or abdomen.

He required continuous positive airway pressure for 36 h and was treated with ampicillin for 48 h. On the third day after birth, he was diagnosed with hypocalcemia, vitamin D deficiency, and hypomagnesemia, and was treated with intravenous calcium and magnesium and oral vitamin D. However, his blood calcium levels remained low, fluctuating between 1.32 and 1.45 mmol/l (normal: 2.11–2.52 mmol/l). On the fifth day, he was treated with intravenous calcium supplementation combined with oral calcitriol (0.1 µg/kg/d), after which serum calcium levels normalized. On the seventh day, calcium and calcitriol were administered orally. Two weeks after birth, serum 25-hydroxyvitamin D increased to normal levels. At the time of discharge at 16 days old, he failed a neonatal hearing test (otoacoustic emission and brainstem auditory response).

**Fig. 2:**
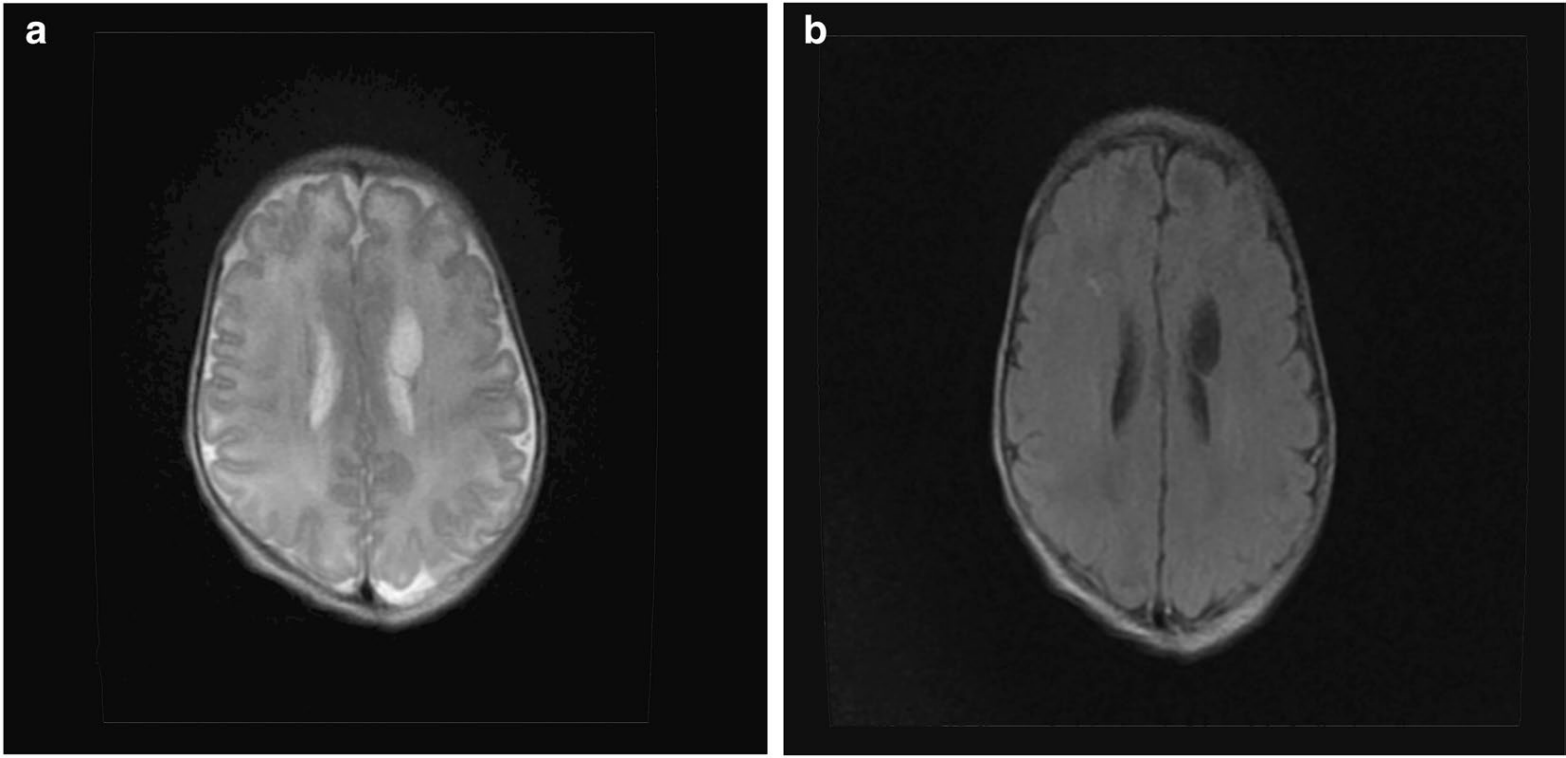
Brain MRI showing a left frontal lobe cyst

Laboratory examination included an electrolyte test on the third day that revealed the following: serum calcium: 1.18 mmol/l (normal 2.11–2.52 mmol/l), magnesium: 0.50 mmol/L (normal: 0.53–1.11 mmol/l), and phosphorus: 2.81 mmol/l (normal: 0.85–1.51 mmol/l). Serum parathyroid hormone levels on the fifth day were 2.8 pmol/l (normal 1.6–6.9 pmol/l), and 25-hydroxyvitamin D levels were 14.90 ng/ml (normal > 25 ng/ml). Brain magnetic resonance imaging (MRI; 1.5 T) performed on the 14th day revealed a malformation cyst in the left frontal lobe (Fig. 2a). Lumbosacral MRI was normal (Fig. 2b). Ultrasound of the scrotum revealed bilateral cryptorchidism, and ultrasound of the urinary system showed absence of the right kidney. At the age of 3 months, he failed a short-sound auditory brainstem response test, confirming binaural sensorineural hearing loss.

During follow-up, hearing loss and psychomotor retardation were noted. He was unable to laugh or control his head at the age of 5 months. On the basis of this observation, a motor evaluation was performed, resulting in an Alberta Infant Motor Scale score of 16 points (20th centile). Long-term follow-up visits showed that serum calcium and vitamin D levels were maintained within normal ranges. He is currently 6 months old (corrected gestational age: 5 months), and still unable to turn over or sit without help. His weight is 6.30 kg (3rd ~ 10th centile), his body length is 66.5 cm (10th ~ 50th centile), and his head circumference is 41.0 cm (3rd ~ 10th centile). He is scheduled for continued physical therapy and routine follow-up by pediatrician.

### Genetic testing

**Fig. 3:**
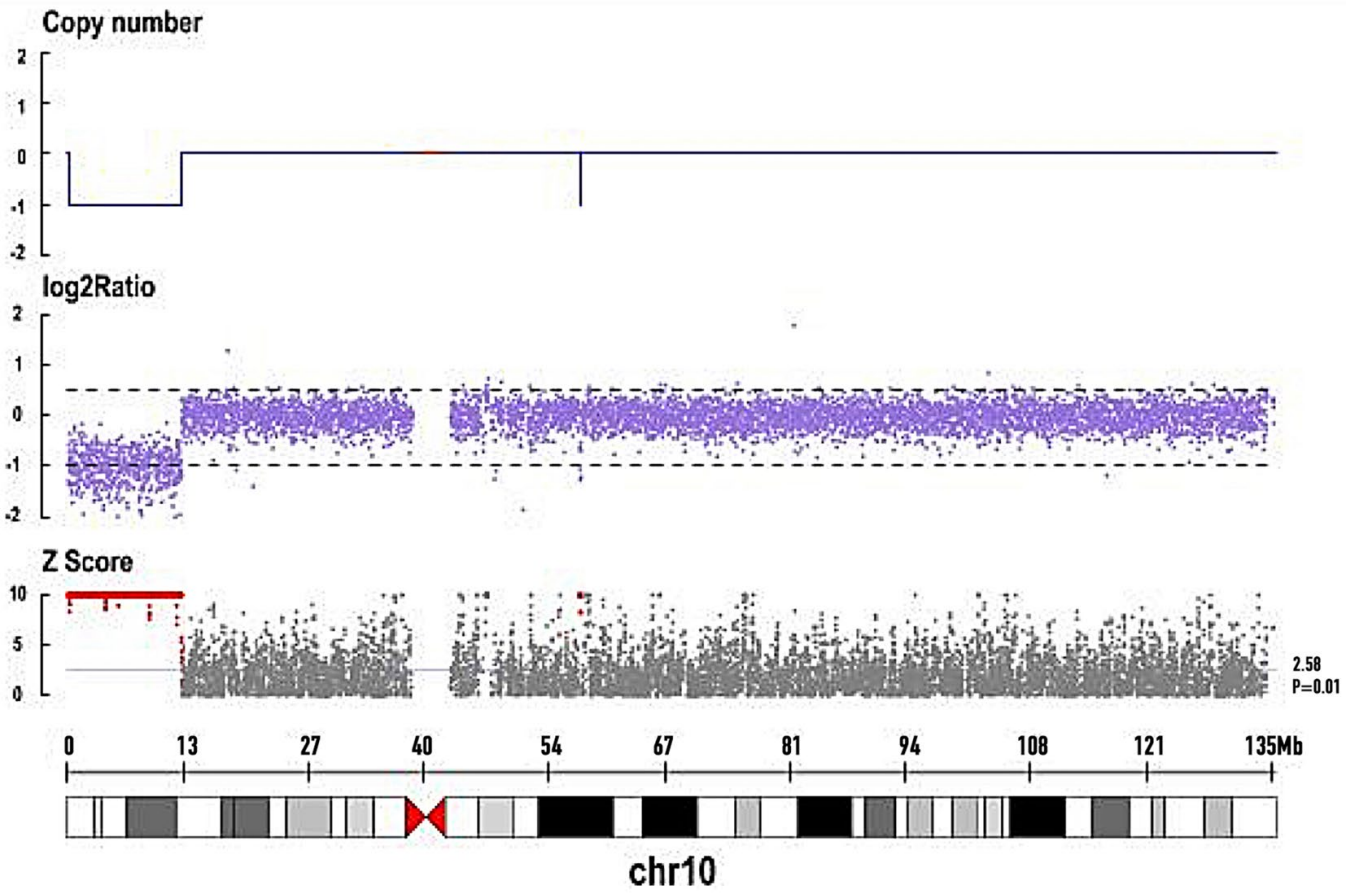
Distribution of chromosome 10 copy numbers in the proband

**Fig. 4:**
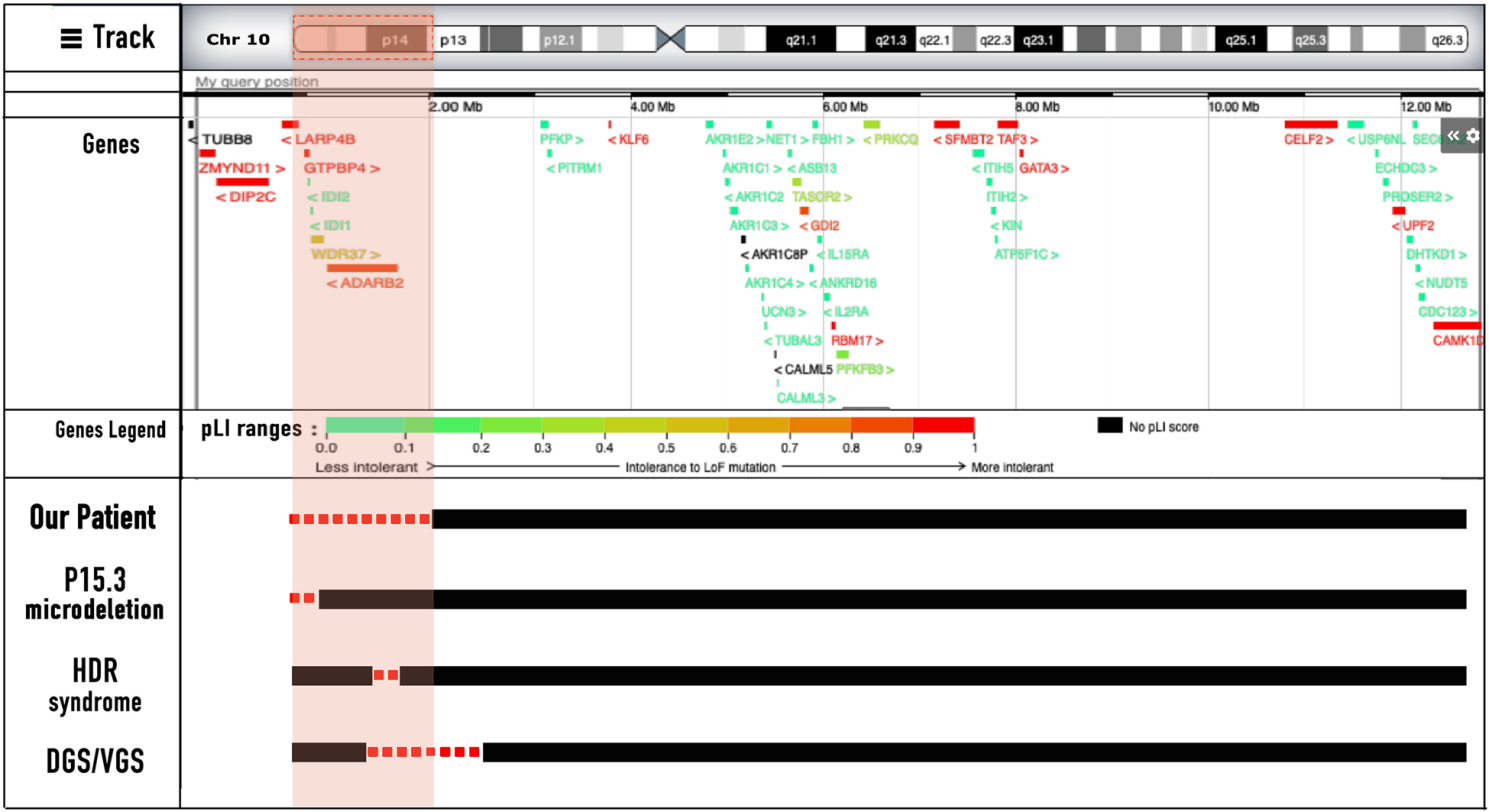
Size of the deletion in patients with partial monosomy 10p

This research was approved by the Ethics Committee of the First Affiliated Hospital of Fujian Medical University, and the patient’s parents provided their written informed consent. Whole blood was used to extract genomic DNA for genetic testing using the TIANamp blood genomic DNA kit (Tiangen, Beijing, China). Genome-wide copy number variation (CNV) was detected by DNA sequencing using the Illumina HiSeq series sequencer (Illumina, San Diego, CA) and analyzed by the cloud platform system using the human population CNV database, OMIM database, and PubMed database. CNV analysis revealed a pathogenic deletion about 12.71 Mb in size in chr10p15.3p13 (chr10: 105,001–12,815,001) (Fig. 3). The deletion involved the *ATP5C1*, *IL2RA*, *DHTKD1*, *KLF6*, *AKR1C2*, *AKR1C4*, *ZMYND11*, and *GATA3* genes (Table 1). The genetic tests of the parents were normal (Fig. 4).

**Table 1:** ResSeq genes within the deleted region (chr10:105,001–12,815,001)

ResSeq genes	OMIM phenotype	MIM number
*ZMYND11*	Mental retardation, autosomal dominant	#616,083
*IL2RA*	Immunodeficiency with lymphoproliferation and autoimmunity	#601,942
*GATA3*	Hypoparathyroidism, sensorineural deafness, and renal dysplasia	#146,255
*DHTKD1*	2-aminoadipic 2-oxoadipic aciduria Charcot-Marie-Tooth disease, axonal, type 2Q	#204,750
*KLF6*	Gastric cancer, somatic #613,659 Prostate cancer, somatic	#613,659
*AKR1C2*	46, XY sex reversal	#614,279
*AKR1C4*	46, XY sex reversal	#614,279

## Discussion and Conclusions

We describe herein a case with terminal 10p deletions presenting with specific facial features, hypoparathyroidism, sensorineural deafness, renal abnormalities, and developmental retardation. Hypoparathyroidism accompanied with dysmorphic features in this newborn were the reasons for performing chromosome analysis. We used CNV detection to analyze the whole genome chromosomal copy number of this patient. The results showed that our patient presented with a terminal deletion in the 10p15.3p13 region, involving *ZMYND11*, *GATA3*, and a critical region of *DGS2*.

Chromosome 10p terminal deletions have been described in more than 50 cases, since the first observation by Elliott et al. [12]. in 1970. *GATA3* deletion is associated with hypoparathyroidism, sensorineural hearing loss, and nephrotic syndrome, while heterozygous loss of *ZMYND11* is responsible for specific facial features and mental retardation. Additionally, the partial deletion of key regions of *DGS2* may cause DiGeorge anomaly, also known as DiGeorge syndrome 2 (DGS2), and characterized by congenital heart malformation, thymus dysplasia, and T cell defects [13].

We reviewed the previous literatures on terminal deletion of chromosome 10, and compared the clinical phenotypes and genotypes of chromosome 10 terminal deletion (see Table 2). In order to avoid the overlap of clinical phenotypes caused by different genovariation, reports with other variations or unclear variations were not included in the summary. Only the proband information was included in the cases with family disease. A total of 18 cases of chromosome 10 terminal deletion were included. In all the cases reviewed, more than half of the patients had specific facial anomalies, including a prominent forehead, broad nasal bridge, low-set ears, and micrognathia. Most of patients showed a wide range of psychomotor retardation, including hypotonia, seizures or autism. More than two-thirds of patients had HDR syndrome. Among them, there were 12 patients with hypoparathyroidism, 10 patients with sensorineural hearing loss, 9 patients with renal or urinary system abnormalities, and 6 patients with the HDR triad. Approximately one-third of the patients had congenital heart disease, which was the major cause of death within the first months of life. (patient17, 18 and patient 19). Urinary tract anomalies were found in approximately one-third of the patients. Frontal lobe cyst, dilatation of cerebral ventricles, and hypoplasia of brainstem were presented in some patients. However, a sacrococcygeal mass, which was observed in our patient, has not been described in any of the other published patients. By comparing the clinical features of our patients with the previously reported patients with pure 10p terminal deletion, we found that the size of the 10p terminal deletion is the most important influence on the clinical phenotype of our patients.

**Table 2:** Phenotypic and genotypic characteristics of present case and previously reported cases with terminal 10p deletions

Patient		10p aberration	Genomic coordinates	Size	Gender	Age at Onset	Age at Diagnosis	HDR spectrum	DGS/VCFS spectrum		Other dysmorphic features
Our Patient	Present case	del(10)(p15.3-p13)	Chr10: 105,001–12,815,001	12.6 MB	Male	3 days	1 month	HDR	Specific facial feature		Mental retardation,delayed motor function, frontal lobe cyst, cryptorchidism
Patient 1	Nina Marić et al. [4]	del(10)(p13)	NR	NR	Female	Neonatal period	Neonatal period	HDR	Specific facial feature		NR
Patient 2	Vidavalur R et al. [22]	del(10)(p15.3p14)	NR	NR	Reference Male	Neonatal period	Neonatal period	HD	Cardiac anomalies		NR
Patient 3	Saet Byeol Kim et al. [23]	del(10)(p15.3-p13)	Chr10: 100,047–16,314,195	16 Mb	Female	Neonatal period	Neonatal period	HDR	Cardiac anomalies	Thymus Hypoplasia/aplasia	Brain anomalies, hypotonia
Patient 4	Birute Tumiene et al. [18]	del(10)(p15.3)	10p15.3 del (102,539–4,440,292)	4.4 Mb	Male	Neonatal period	7 months	NR	Specific facial feature		Muscular hypotonia, psychomotor retardation, laryngotracheomalacia
Patient 5	Bruno F. Gamba et al. [5]	del(10)(p15.3p14)	Chr10: 2,834,518–8,485,795	NR	Male	Neonatal period	3 months	NR	Bilateral cleft lip/palate		Delayed psychomotor development
Patient 6	Daniela Melis et al. [3]	del(10)(p14)	Chr10: 7,914,774–12,105,010	4.19025 Mb	Male	NR	4 years	H	NR		Psychomotor retardation, palpebral ptosis, epicanthic folds, anteverted nares, cryptorchidism, hand/foot abnormalities
Patient 7	Cheryl DeScipio et al^.^[14]	del(10)(p15.3)	chr10:214,559- 2,464,948	2,250,389 bp	Male	NR	10 years	R	NR		Mild intellectual disability, developmental articulation disorder, language disorder, hypospadia
Patient 8	Cheryl DeScipio et al. [14]	del(10)(p15.3)	chr10: 62,797- 1,230,967	2,250,389 bp	Male	NR	5 years	NR	NR		Craniofacial dysmorphism, partial complex seizures, diaphragmatic hernia, and developmental, motor and language delay
Patient 9	Cheryl DeScipio et al. [14]	del(10)(p15.3)	chr10:115,544–1,748,581	1,633,037 bp	Male	NR	8 years	NR	NR		craniofacial dysmorphism, developmental delay, autistic behavior, stereotypy and developmental and speech delay
Patient 10	Cheryl DeScipio et al. [14]	del(10)(p15.3)	chr10: 106,829- 3,812,917	3,706,088 bp	Male	NR	38 years	NR	NR		craniofacial dysmorphism, growth delay, moderate intellectual disability, global psychomotor delay
Patient 11	Elisa Benetti et al. [6]	del(10)(p15-p12)	NR	NR	Female	8 month	15 months	DR	NR		Epilepticus, growth and mental retardation
Patient 12	Annapia Verri et al. [7]	del(10)(p15-p14)	NR	NR	Male	2 months	33 years	HR	Specific facial feature		Developmental delay, Autism
Patient 13	Peter Lichtner et al. [2]	del(10)(p13)	WI-2389	NR	Female	Neonatal period	7 years	HDR	Specific facial feature		Developmental delay
Patient 14	Peter Lichtner et al. [2]	del(10)(p13)	D10S1720	NR	Female	Neonatal period	12 years	HDR	Specific facial feature		Developmental delay
Patient 15	Majed Dasouki et al. [8]	del(10)(p13)	D10S547	NR	Male	Neonatal period	15 years	HD	Specific facial feature, T cell abnormality		Epilepticus, growth and mental retardation
Patient 16	Moshe Shapira et al. [9]	del(10)(p13)	NR	NR	Male	Neonatal period	4 years	DR	Specific facial feature		Developmental delay, psychomotor retardation
Patient 17	R.Berger et al. [10]	del(10)(p13)	NR	NR	Male	Neonatal period	Neonatal period	H	Cardiac anomalies, Specific facial feature		Small penis, left undescended testis, elub hands and club feet. dehiscent abdominal muscular wall
Patient 18	M. H. Shokeir et al. [11]	del(10)(p13)	NR	NR	Male	Neonatal period	NR	NR	Cardiac anomalies, Specific facial feature		NR

HDR, hypoparathyroidism (H), sensorineural hearing loss (D), and renal dysplasia (R); NR, no reported;Del, deletion; P, patient;

The deletion of the 10p15p13 terminal fragment, identified in our patient, is closely related to 10p15.3 microdeletion syndrome which is associated with neurodevelopmental disorders and characterized by developmental retardation, intellectual disability, craniofacial deformity, behavioral abnormalities, hypotonia, and seizures [14]. Mutations in *ZMYND11* are reported to be causative for 10p15.3 microdeletion syndrome [15]. Coe et al. [16] created an expanded CNV morbidity map from 29,085 children with developmental delay in comparison with 19,584 healthy controls, and pinpointed the haploinsufficiency of *ZMYND11* as being associated with subtle facial dysmorphism, mild intellectual disability, and neuropsychiatric behavioral features. Moskowitz et al. [17] identified *ZMYND11* heterozygous variants in patients with specific facial features, autism spectrum disorders, mental retardation, aggression, and complex neuropsychiatric characteristics, supporting the association of *ZMYND11* with 10p15.3 deletion syndrome. Moreover, Tumiene et al. [18] compared the clinical phenotypes of 14 patients with 10p15.3 deletions with the phenotypes of eight patients with loss-of-function *ZMYND11* mutations, then further confirmed that *ZMYND11* was the critical gene for the 10p15.3 microdeletion clinical phenotype. Our patient presenting with the entire deletion of *ZMYND11* gene, has specific facial features that are consistent with 10p15.3 microdeletion clinical phenotype. Although still young, he has already developed psychomotor retardation. Therefore, we should continue to follow up his future neuropsychiatric development.

Our patient carries a *GATA3* deletion in the 10p15.3p13 region, accompanied by hypoparathyroidism, sensorineural deafness, and renal dysplasia, and was diagnosed with HDR syndrome, also known as Barakat syndrome.The main clinical features of HDR syndrome are the triad of hypoparathyroidism (H), sensorineural hearing loss (d) and renal dysplasia (R). The other clinical features of HDR syndrome are variable, including hypomagnesemia and vitamin D deficiency, insulin-dependent diabetes mellitus, congenital heart disease, specific facial features, cerebral infarction, severe cognitive impairment and autism [19]. In 2000, Van Esch et al. [20] found that *GATA3* was essential for embryonic development of the parathyroids, auditory system, and kidneys, and also revealed that *GATA3* haploinsufficiency causes human HDR syndrome. To date, several types of *GATA3* mutations related to HDR syndrome have been reported in nearly 124 families (177 patients) worldwide. These include about 40% frameshift deletions or insertions, 23% missense mutations, 14% nonsense mutations, 6% splice site mutations, 1% in-frame deletions or insertions, 15% complete gene deletions, and 1% complete gene duplications [21]. Vidavalur et al. [22] reported a male premature infant who was small for gestational age, with micrognathia and facial malformation, combined with hypoparathyroidism and bilateral sensorineural hearing loss. CNV examination revealed the absence of chromosome 10p (10p15.3p14), including *GATA3* and other genes. This further confirmed that *GATA3* is a critical gene for HDR syndrome. Our patient presents with a typical triad of hypoparathyroidism, sensorineural hearing loss, and renal dysplasia, and we suggest that his *GATA3* deletion is the main cause of the disease.

Our patient was found to have partial monosomy for a proximal deletion of chromosome 10p14-p13, which is associated with DGS2. Typical DGS is caused by the microdeletion of chromosome 22q.12.2. However, in 1996, Daw first stated that haploinsufficiency of a gene or genes within chromosome 10p (the *DGS2* locus) can cause DGS spectrum, defined as DSG2 (OMIM #601,362). Lichtner et al. [2] suggested that the DGS phenotype associated with a 10p deletion should be considered a contiguous gene syndrome owing to terminal deletions between D10S585 and D10S1720. Hemizygosity of the proximal region, designated *DGCR2*, can cause cardiac defects and T cell deficiency. In 2017, Kim et al. [23] reported a female infant (patient 3) with the largest deletion of the chromosome 10p, presenting with hypoparathyroidism, hearing loss, cardiac abnormalities, thymus hypoplasia, a double uterus, double cervix, and relatively small kidneys. Unexpectedly, our patient has so far only presented with a few clinical features of DGS2 (psychomotor retardation and cryptorchidism) and lacks other typical signs such as cardiac defects, a cleft palate, thymus dysplasia, and abnormal T cell levels. The mechanism for this is still unclear, but it may be related to the different effects of large fragment deletions on individuals and gene mutations. Some specific manifestations involving 10p13- p14 have yet to be determined as the patient is still very young; however, an adverse clinical course is to be expected.

In conclusion, the combined data from the present case and previous reported cases support that the terminal deletion of chromosome 10p can be considered as a contiguous gene syndrome, related to a variety of clinical characteristics including specific facial features, psychomotor retardation, hypoparathyroidism, sensorineural hearing loss, and renal dysplasia. In contrast to previously reported cases, our patient was diagnosed early in the neonatal period, which has been very important for early clinical intervention. For patients with similar clinical manifestations, we suggest that genetic analysis should be performed to identify the precise molecular defects to further elucidate the genotype–phenotype correlation of chromosome 10p deletions.

## Data Availability

All data generated in this study are included in this published article.

## Abbreviations

DGS: DiGeorge syndrome
MRI: Magnetic resonance imaging
CNV: Copy number variation
DNA: Deoxyribonucleic acid
OMIM: Online Mendelian Inheritance in Man
HDR: Hypoparathyroidism, deafness and renal dysplasia

## References

[1] Schuffenhauer S, Lichtner P, Peykar-Derakhshandeh P (1998). Deletion mapping on chromosome 10p and definition of a critical region for the second DiGeorge syndrome locus (DGS2). Eur J Hum Genet.

[2] Lichtner P, König R, Hasegawa T, Van Esch H, Meitinger T, Schuffenhauer S (2000). An HDR (hypoparathyroidism, deafness, renal dysplasia) syndrome locus maps distal to the DiGeorge syndrome region on 10p13/14. J Med Genet.

[3] Melis D, Genesio R, Boemio P, Del Giudice E, Cappuccio G, Mormile A, Ronga V, Conti A, Imperati F, Nitsch L, Andria G (2012). Clinical description of a patient carrying the smallest reported deletion involving 10p14 region. Am J Med Genet A.

[4] Mari N (2018). Terminal deletion of chromosome 10p13 as a cause of hypoparathyroidism in a neonate. Central Eur J Paed..

[5] Gamba BF, Rosenberg C, Costa S (2015). Cleft lip/ palate, short stature, and developmental delay in a boy with a 5.6-Mb interstitial deletion involving 10p15.3p14(Article). Molecul Syndromol.

[6] Benetti E, Murer L, Bordug A (2009). 10p121 deletion: HDR phenotype without DGS2 features. Exp Mole Pathol.

[7] Verri A, Maraschio P, Devriendt K (2004). Chromosome 10p deletion in a patient with hypoparathyroidism, severe mental retardation, autism and basal ganglia calcifications. Ann Genet.

[8] Dasouki M, Jurecic V, Phillips JA (1997). DiGeorge anomaly and chromosome 10p deletions: One or two loci?. Am Med Genet.

[9] Shapira M, Borochowitz Z, Bar-El H (1994). Deletion of the short arm of chromosome 10 (10p13): report of a patient and review. Am J Med Genet.

[10] Berger R, Larroche JC, Toubas PL (1977). Deletion of the short arm of chromosome no 10. Acta Paediatr Scand.

[11] Shokeir MH, Ray M, Hamerton JL (1975). Deletion of the short arm of chromosome No 10. J Med Genet.

[12] Elliott D, Thomas GH, Condron CJ (1970). C-group chromosome abnormality (10p-) Occurrence in a child with multiple malformations. Am J Dis Child.

[13] Anderlid BM, Schoumans J, Nordgren A, Malmgren H, Verri A, Blennow E, Eriksson M, Lindstrand A, Benetti E, Golovleva I (2010). Molecular and clinical characterization of patients with overlapping 10p deletions. Am J Med Genet A.

[14] DeScipio C, Conlin L, Rosenfeld J (2012). Subtelomeric deletion of chromosome 10p153: clinical findings and molecular cytogenetic characterization. Am J Med Genet A.

[15] Hong Wen, Yuanyuan Li, Haitao Li, et al. ZMYND11: an H3.3-specific reader of H3K36me3. Cell cycle (Georgetown, Tex), 2014, 13(14):2153–2154. 10.4161/cc.29732 24963723

[16] Coe BP, Witherspoon K, Rosenfeld JA (2014). Refining analyses of copy number variation identifies specific genes associated with developmental delay. Nat Genet.

[17] Moskowitz AM, Belnap N, Siniard AL (2016). A de novo missense mutation in ZMYND11 is associated with global developmental delay, seizures, and hypotonia. Cold Spring Harb Mol Case Stud.

[18] Tumiene B, Čiuladaitė Ž, Preikšaitienė E (2017). Phenotype comparison confirms ZMYND11 as a critical gene for 10p15.3 microdeletion syndrome(Article). J Appl Genet.

[19] Barakat AJ, Raygada M, Rennert OM (2018). Barakat syndrome revisited. Am J Med Genet A.

[20] Van Esch H, Devriendt K (2011). Human genome and diseases: review. Transcription factor GATA3 and the human HDR syndrome. Cell Mol Life Sci.

[21] Lemos MC, Thakker RV (2020). Hypoparathyroidism, deafness, and renal dysplasia syndrome: 20 Years after the identification of the first GATA3 mutations. Hum Mutat.

[22] Vidavalur R, Devapatla S (2018). A Unique Genomic Variant of HDR Syndrome in Newborn. Indian Pediatr.

[23] Kim S, Kim Y, Jung J (2017). Clinical description of a neonate carrying the largest reported deletion involving the 10p15.3p13 region. Clin Case Rep.

